# The Dutch version of the Dignity Therapy Question Protocol for individual Dutch nursing home residents without cognitive impairments (during COVID-19): a pilot study

**DOI:** 10.1186/s12877-024-05632-8

**Published:** 2025-01-06

**Authors:** Herman van Dammen, Kris Vissers, Gert-Jan van der Putten, Yvonne Engels

**Affiliations:** 1 ¹Department of Anesthesiology, Pain and Palliative Medicine, Radboud University Medical Center, Nijmegen, The Netherlands; 2 ²Dental Department, Radboud University Medical Center, Nijmegen, The Netherlands; 3 Orpea Dagelijks Leven, Apeldoorn, The Netherlands

**Keywords:** Nursing home residents, Dignity therapy question protocol, DTQP, D-DTQP, Translation, Feasibility

## Abstract

**Background:**

Dignity therapy is a brief, structured psychotherapeutic intervention originally designed to help last-stage cancer patients maintain their dignity. It consists of a semi-structured interview encouraging patients to talk about their lives. The recorded session are transcribed and edited, after which the patient has the opportunity to make further changes to the final document. It can be shared with whom the patient likes. We cross-culturally translated dignity therapy into Dutch and explored its feasibility of applying it to Dutch cognitively capable nursing home residents.

**Method:**

Beaton’s Guidelines for Good Clinical Practice were used to cross-culturally translate dignity therapy. Next, a pilot study was conducted with 12 nursing home residents in which the original English-language questionnaire was transculturally translated according to the method of Beaton. After the interviews were completed, feasibility was examined by open-ended questions and a five-point Likert scale questionnaire.

**Results:**

We translated the questionnaire. The translated questions were well understood and resulted in an appropriate Dutch version of the English-language dignity therapy questions. However, the words ‘dignity’ and ‘therapy’ caused confusion. The mean number of words in the legacy document was 1078 words, which appeared shorter than in a community-based hospice setting or patients with metastatic cancer or terminally ill people. The reading aloud was much appreciated and was an emotional experience for some.

**Conclusion:**

We successfully translated the DTQP into the Dutch language and tested it in 12 nursing home residents. The questions posed by this therapy appeared suitable and acceptable. Furthermore, participants all accepted the therapy and gave no suggestions to adapt the procedure. Follow-up research in the form of an impact study is needed to show whether and how this therapy can strengthen the sense of dignity of Dutch nursing home residents.

## Background

Nursing home residents are vulnerable to losing their personal dignity [1–3] by having moved to an unfamiliar environment, being without their families and having little privacy. In addition, due to their increasing fragility and progressive social isolation, they are highly dependent on the continuous support of dedicated nursing staff and informal caregivers [4]. Furthermore, disease-related conditions can induce existential stress and loss of dignity [5]. The current shortage of nursing staff poses an additional threat to preserving their dignity [6, 7], with less time being available to help with social interaction, toileting, daycare and personal care [8].

Dignity is a subjective and multidimensional concept. Personal dignity is a type of dignity that relates to a sense of worthiness, is individualistic, tied to personal goals and social circumstances, and can be taken away or enhanced by circumstances or acts from others [4]. Chochinov developed an empirical model in which he distinguishes three main categories of personal dignity: illness-related concerns, dignity-conserving repertoire and social dignity inventory. These three categories in turn are subdivided into themes and subthemes, all influencing personal dignity [9] as seen in Fig. 1 in Appendix 1. Individuals whose dignity is affected or lost can perceive depression, the will to live or feelings of being abandoned [9–11]. Their quality of life is seriously threatened [12]. Consequently, maintaining and improving the dignity and quality of life of nursing home residents should be a priority of management and healthcare professionals of nursing homes [13].

Dignity therapy, originally developed for patients in the final stage of cancer, is a short, structured psychotherapeutic intervention. It displays satisfactory effects and has been recommended for clinical and epidemiologic research [14, 15]. Dignity therapy starts with an interview with a trained healthcare professional, which is based on the themes and subthemes of Chochinov’s empirical model. The answers are transformed into a written narrative, which in a next meeting is read aloud to the resident. The resident receives this document, which he or she is free to share with others as a legacy document. In patients with advanced cancer, the Dignity therapy intervention improved significantly and clinically relevant dignity [16]. It gives a patient the chance to record meaningful aspects of his or her life and to leave something behind that can benefit their loved ones in the future.

Dignity therapy might also be helpful to conserve nursing home residents’ sense of dignity by addressing sources of psychosocial and existential distress [16, 17]. Even though it has been translated into several languages like Danish [18], German [19] and Italian [20], a Dutch translation was not available. Because of variations in social and economic structures, culture and language, health questionnaires must be adapted cross-culturally to retain the psychometric properties of the original version.

Therefore, the aims of the present study are (1) to cross-culturally translate the Dignity Therapy Question Protocol (DTQP) and (2) to evaluate its feasibility among Dutch nursing home residents.

## Methods

### DTQP translation

#### Design

The English version of the DTQP is presented in Appendix 2. For the translation and cultural adaptation process, we followed the “Guidelines for the Process of Cross-Cultural Adaptation” [21]. The translation was conducted by two people with Dutch as their mother tongue, of which one had knowledge of the subject (GvdP, PhD elderly care physician) and the other had no knowledge of it (JV, PhD in agricultural science). Subsequently, a consensus translation was made with an independent third party (AW, MSc MA PhD).

Then, two native English speakers (MH and DH) also mastered Dutch, each independently back-translated the Dutch consensus version to English. They did not know the original questionnaire. Next, an expert panel compared and synthesised the translated and back-translated versions into one version. This expert panel consisted of three specialists in meaningful healthcare and palliative care (YE; GvdP; KV), all fluently speaking both English and Dutch. They compared the conceptual equivalence (conceptual meaning to terms and concepts in the Dutch population), clarity (understandable expressions), and popularity (avoidance of technical terms) between the two versions. Then, a preliminary Dutch DTQP version was produced.

The Dutch version of the DTQP is presented in Appendix 3.

### Feasibility study

#### Design

We aimed to test the feasibility of the preliminary version of the Dutch DTQP in 12 nursing home residents.

Residents of four nursing homes in the centre of the Netherlands were asked to participate in this part of the study. Healthcare professionals were asked to identify potential residents. The inclusion criteria of residents were being 60 years of age or older and cognitively capable of participating, as indicated by an involved healthcare professional (psychologist, nurse, physician). In case of any ambiguity of cognitive capability, the Mini-Mental State Examine MMSE-2 short version was applied by a staff member (professional caregiver or psychologist) to examine their cognitive status. If residents had a T score ≤ 30 (2 standard deviations or more below the mean), they were excluded from the study. Other exclusion criteria were not being able to communicate (due to, for example, aphasia), not understanding the instructions and questions, being too ill to participate or not being able to decide independently about participation (this last item was determined by the head of the department).

Potential participants were given verbal information about the study, supported by detailed written information. The questions of the DTQP were added to this written information so that they already knew what kind of questions would be asked. Three to five days later, the person was asked if they wished to participate in the study, and if they agreed, informed consent was signed.

### Data collection

The DTQP was performed by the researcher/psychologist (HvD), who had followed a 2½-day interactive dignity therapy workshop led by Dr. Harvey Max Chochinov in Winnipeg Canada.

After an appointment was made, the questions were presented to the participant during an interview. The psychologist followed the participant’s train of thought, inquired and followed the protocol. The interview was audio-recorded, transcribed and edited into a coherent narrative. After each interview, the interviewer wrote a short evaluation of the process of the interview.

Within 1–2 weeks after this visit, the researcher visited the participating resident again. During this meeting, he read aloud the narrative to the resident. If needed by the resident, textual changes were made, after which the document, in an attractive layout, was made permanent and given to the resident as a “generativity” or “legacy” document. Immediately afterwards, feasibility was tested by a questionnaire with questions about the comprehensibility, acceptability, and relevance of the questions. To assess the feasibility of the dignity therapy, a five-point Likert scale (fully agree-fully disagree) was used with questions such as: The questions asked in the interview were understandable, The questions I was asked were relevant to me, It was difficult to answer the questions, The questions asked in the interview were understandable, I enjoyed being interviewed. The sum of fully agree and agree together was used to report feasibility. Another question was whether the participant shared the document with others (yes or no). Finally, two open-ended questions were asked: How did you feel about the length of the interview, and what could be done differently?

For each participating resident, additional data, such as date of admission, date of birth, sex, and main diagnosis, were retrieved from the medical records. All interviews were recorded, transcribed verbatim, edited and then reshaped into a narrative.

The process of testing feasibility took place from August 2020 until April 2021, during the COVID-19 pandemic. In this period, nursing home residents had limitations in receiving relatives and had to stay strictly in their rooms. Protective means had to be used by themselves and the nursing staff [22].

## Results

### Characteristics of the participants

Fourteen residents (10 women and 4 men) were invited to participate. One resident declined because she found it too confronting to tell her life story. Another resident first agreed to participate, but after the interview had been recorded, transcribed, and given back to the resident for possible adjustments or corrections, this resident, after having read the document, decided to discontinue. The participant concluded that she had told too much of her life story and continued to have problems with the concept of dignity concerning her stay in the nursing home. Of the remaining 12 residents, the mean age was 77 years (range 69–90). The main diagnoses were cerebrovascular accident (CVA) [6], morbid adiposity [1], hypertension [1], polyneuropathy [1], lateral sclerosis [1], Parkinson’s disease [1] and chronic kidney failure [1]. Most residents had multimorbidity. The mean length of the interview was 21 min (range 8–34). The legacy document had a mean of 1078 words (range 536 to 1655).

### The interviews

Only the resident was present, apart from one interview where the resident wanted the partner to be present as well. In this case, the resident frequently asked for support from her partner in answering the questions. Participants’ answers were usually quite short. Therefore, after seven sessions, more effort was made to achieve more in-depth answers to the question, hoping to gain increased value through more reflection on the initial questions. However, the mean length of the transcripts (respectively 1880 and 2010 words) or the legacy document (respectively 1099 and 1047 words) did hardly differ between interviews 1–7 and 8–12.

The words ‘dignity’ and ‘therapy’ were both perceived as confusing by some participants, and it was also mentioned that some questions resulted in overlapping answers. When the narrative was read aloud and returned, some participants became emotional because of the intensity their own words evoked.

It is striking that several questions, where the interviewer expected longer answers, were answered briefly or ‘superficially’ by our participants.

### Feasibility of the dignity therapy procedure

All participants considered the questions during the interview understandable (12; 100%), and all but one found them relevant (11; 91%). Additionally, all participants considered that the legacy document reflected the important aspects that had been discussed during the interview. (12; 100%) and ten participants would recommend this therapy to others (10; 83%).

When asked what they thought about the interview, words such as ‘interesting’, ‘very pleasant process’ and ‘confronting because you pick up a lot’ were mentioned. All of them found the length of the interview fine.

## Discussion

This feasibility study demonstrates that the Dutch translation of the Canadian Dignity Therapy Question Protocol (DTQP) is useful and applicable for nursing home residents in the Netherlands. The translation was adequate. All but one resident found the intervention suitable and acceptable. All residents highly valued the intervention and the legacy document, which is in line with a systematic review of dignity therapy, with a variety of patient populations [23].

The mean number of words in our legacy documents was approximately 1000, with a spread from 500 to 1500. And even though we put much effort into elaborating more depth in the last five interviews, this did not influence the outcome. Of course, interview outcomes may and will be influenced by the interviewer, but the interviewer in our study is an experienced interviewer and senior psychologist in elderly care. We hypothesize that explaining the interview and handling the interview questions a few days before the interview was a barrier to talking spontaneously during the interview. The answers had already been thought in advance. In other studies, a large spread across the various patient groups exists. In a study on the implementation of Dignity Therapy in a community-based hospice setting, the documents consisted of 1387–6842 words [24]; in a study on patients with metastatic cancer, it contained between 1799 and 11,195 words [25]; and in a study with terminally ill people, these figures were 4605 and 19,763 [26]. The much lower figures in the present study may be partly explained by the fact that we included older residents who live in an environment with less dynamic and social contacts than when you have an advanced, terminal disease at a younger age, who have a family and live at home. Moreover, even though most residents die within two years after admission, contrary to terminally ill patient groups, most of them have not had a clear marking moment where they have heard that they are palliative patients (https://www.verenso.nl/magazine-juni-2022/no-3-juni-2022/wetenschap/korter-verblijf-in-de-langdurige-zorg).

Regarding the brief answering of questions, a few questions were particularly related to legacy. The residents’ answers to questions such as “What have you learned about life that you would like to pass on along to others” and, “What advice or words of guidance do you wish to pass along to your (son, daughter, husband, parents or others)” and “Are there words or perhaps instructions that you would like to offer your family to help prepare them for the future” had a lot of overlap. One of the reasons why, in our study, such questions were answered briefly might be that nursing home residents are less preoccupied with thoughts of nearing the end of life, death and legacy than patients with advanced cancer, for whom dignity therapy was originally developed. Another reason could be the overlap between the different questions, as was also the case in one patient from Houmann’s study [18].

The word dignity, which we (in Dutch) used to introduce the intervention, appeared to confuse the residents of our study. The same applied to the word “therapy”. In its original Greek meaning (θεραπεία) it means “treatment”. However, in Dutch, it is usually used for something that takes several sessions and not a one-time treatment such as dignity therapy. Moreover, when dignity therapy was introduced to the nursing home residents, the idea quickly arose that there was something wrong with them that needed treatment. The confusion concerning the word dignity is in line with a Danish study on dignity therapy, in which five of 20 residents had problems with the title “dignity therapy” [18]. Enes’ research also showed that the word dignity is a complex phenomenon [27].

### Strengths and limitations

It is a strength of our study that we used a proven method to cross-culturally translate the Dignity Therapy Question Protocol. This, in combination with pilot testing and adapting the entire procedure, has resulted in a feasible Dutch nursing home version of dignity therapy.

Given that we managed to complete the study during the COVID-19 pandemic, it seems to indicate the importance of the topic: health care professionals who play a major role in identifying potential residents were willing to include residents, and residents were willing to participate in a threatening, anxious and uncertain period.

If the study had not taken time during the lockdown of COVID-19, we would have chosen to include more participants. However, as the study took place during the COVID-19 lockdown, we beforehand determined a feasible number of 12 participants, taking the COVID-19 restrictions which certainly influenced accrual into account. The same goes for the fact that the interviews were conducted by just one specially trained researcher. In this regard, it may have been an advantage that the researcher also works as a psychologist and, in addition to the dignity therapy training, was also able to employ learned communication skills. In follow-up research, it would be desirable to have more persons conduct the interview to further improve the method. This small study included two individuals who did not participate. A larger study will provide more insight into what the reasons may be.

Finally, in this pilot study, we could not control for possible other influencing factors such as setting or the fact that it was during the pandemic.

## Conclusions

We successfully cross-culturally translated to the Dutch language and tested the DTQP in 12 nursing home residents. The questions posed by this therapy appeared suitable and acceptable. Furthermore, participants all appreciated the therapy and gave no suggestions to adapt the procedure. However, the term dignity therapy appears to raise some questions in the Dutch-speaking world.

Follow-up research in the form of an impact study is needed to show whether and how this therapy can strengthen the sense of dignity of Dutch nursing home residents.

## Appendix 1

**Fig. 1:**
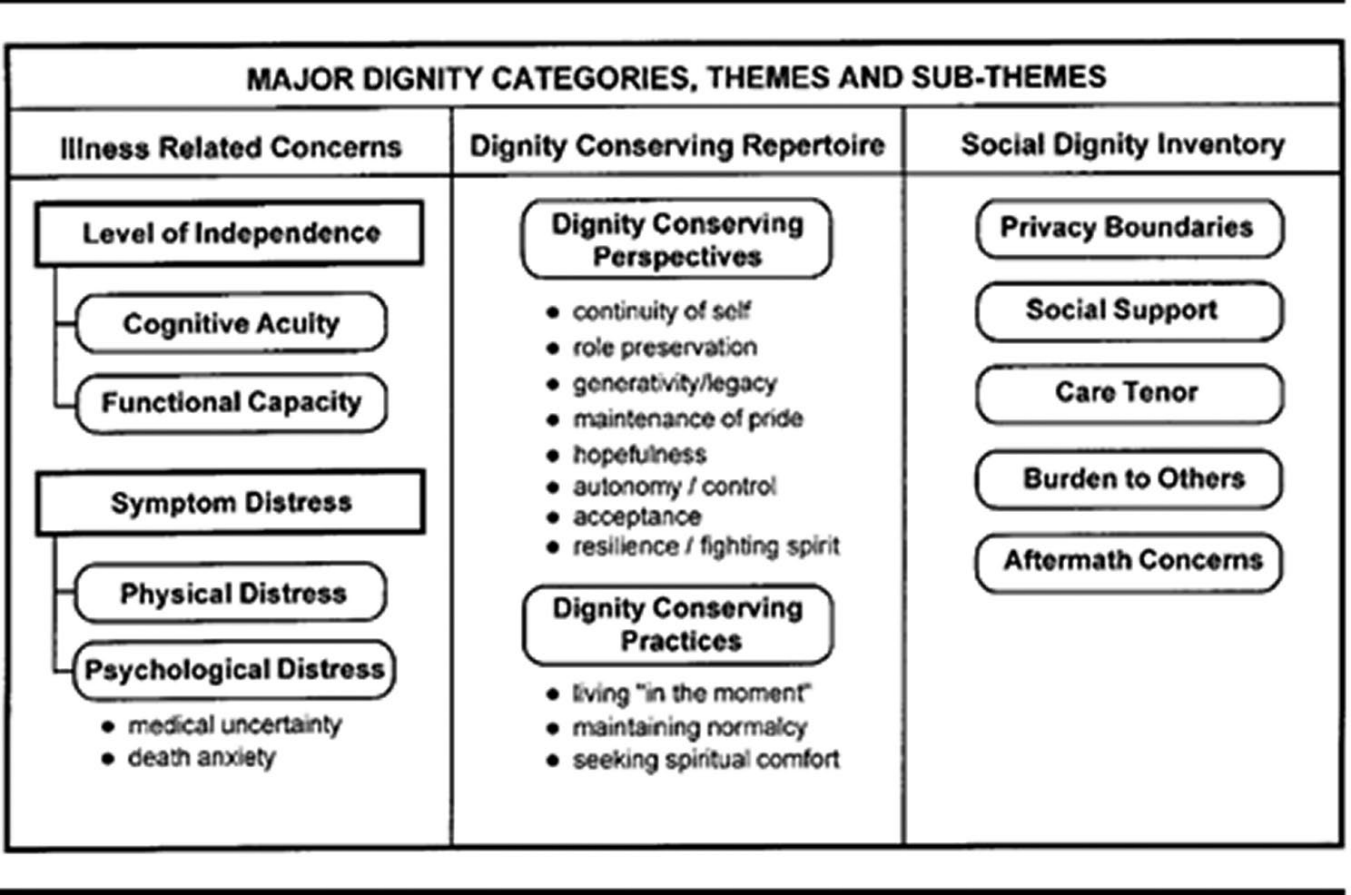
Major dignity categories, themes and subthemes with permission of the developer

## Appendix 2

Original Dignity Therapy Questionnaire (DTQP) with permission of the developer.

- Tell me a little about your life history; particularly the parts that you either remember most or think are the most important. When did you feel most alive?
- Are there specific things that you would want your family to know about you, and are there particular things you would want them to remember?
- What are the most important roles you have played in life (family roles, vocational roles, community-service roles, etc.)? Why were they important to you, and what do you think you accomplished in those roles?
- What are your most important accomplishments, and what do you feel most proud of?
- Are there particular things that you feel still need to be said to your loved ones or things that you would want to take the time to say once again?
- What are your hopes and dreams for your loved ones?
- What have you learned about life that you would want to pass along to others?
- What advice or words of guidance would you wish to pass along to your (son, daughter, husband, wife, parents, other[s])?
- Are there words or perhaps even instructions that you would like to offer your family to help prepare them for the future?
- In creating this permanent record, are there other things that you would like included?

## Appendix 3

The Dutch Dignity Therapy Question Protocol (D-DTQP).

- Kunt u me wat vertellen over uw levensgeschiedenis; met name de delen die u het best herinnert of die voor u het belangrijkst zijn. Wanneer of op welk moment leefde u het meest intens?
- Zijn er bepaalde gebeurtenissen/dingen over u waarvan u graag wilt dat uw familie ze weet, en zijn er bepaalde zaken waarvan u wilt dat zij zich die herinneren?
- Wat zijn de belangrijkste rollen die u in uw leven heeft vervuld (b.v. in de familie, beroepsmatig of in vrijwilligerswerk). Waarom was iedere rol zo belangrijk voor u en welke betekenis had iedere rol voor u?
- Wat zijn uw belangrijkste prestaties, en waar bent u het meest trots op of over?
- Zijn er bepaalde dingen waarvan u het nodig vindt dat u ze tegen uw dierbaren zegt, of dingen waarvoor u de tijd wilt nemen om ze nogmaals te zeggen?
- Wat wenst u in uw dromen voor de mensen die u dierbaar zijn?
- Wat heeft u over het leven geleerd dat u aan anderen door zou willen geven? Welk advies of behulpzame/wijze woorden zou u willen meegeven aan mensen die belangrijk zijn in uw leven?
- Zijn er belangrijke woorden of een boodschap die u wilt meegeven aan uw familie?
- Zijn er nog andere dingen die u graag in dit document opgenomen zou willen hebben?

## Data Availability

The data that support the findings of this study are available on request from the corresponding author. The data are not publicly available due to privacy or ethical restrictions.
